# Laboratory diagnosis of persistent human chlamydial infection

**DOI:** 10.3389/fcimb.2013.00099

**Published:** 2013-12-17

**Authors:** Mirja Puolakkainen

**Affiliations:** ^1^Department of Virology, Haartman Institute, University of HelsinkiHelsinki, Finland; ^2^HUSLAB, Department of Virology and Immunology, Helsinki University Central HospitalHelsinki, Finland

**Keywords:** diagnosis, persistent infection, *Chlamydia pneumoniae*, *Chlamydia trachomatis*, proteomics

## Abstract

Diagnostic assays for persistent chlamydial infection are much needed to conduct high-quality, large-scale studies investigating the persistent state *in vivo*, its disease associations and the response to therapy. Yet in most studies the distinction between acute and persistent infection is based on the interpretation of the data obtained by the assays developed to diagnose acute infections or on complex assays available for research only and/or difficult to establish for clinical use. Novel biomarkers for detection of persistent chlamydial infection are urgently needed. Chlamydial whole genome proteome arrays are now available and they can identify chlamydial antigens that are differentially expressed between acute infection and persistent infection. Utilizing these data will lead to the development of novel diagnostic assays. Carefully selected specimens from well-studied patient populations are clearly needed in the process of translating the proteomic data into assays useful for clinical practice. Before such antigens are identified and validated assays become available, we face a challenge of deciding whether the persistent infection truly induced appearance of the proposed marker or do we just base our diagnosis of persistent infection on the presence of the suggested markers. Consequently, we must bear this in mind when interpreting the available data.

## Background

The clinical spectrum of human chlamydial infections includes clinically unapparent infections, acute symptomatic infections as well as persistent infections (defined as the presence of viable but non-cultivable chlamydiae). Persistent *Chlamydia psittaci* infection in cultured cells was described over 30 years ago (Moulder et al., 1980). Similar continuous infection models in cell lines without external induction have later been established for *Chlamydia trachomatis* (Lee and Moulder, 1981) and *Chlamydia pneumoniae* (Kutlin et al., 1999). In cultured cells, persistent infection can also be induced by external factors, including amino acid starvation, interferon-γ-induced tryptophan deprivation, iron chelation, tobacco smoke and viral co-infection as well as by exposure to antimicrobial agents (for review, see Beatty et al., 1994; Hogan et al., 2003). Furthermore, chlamydial infection in monocyte/macrophage cultures has the appearance of a persistent infection (Mannonen et al., 2004, 2011). The presence of large, pleomorphic reticulate bodies, named aberrant bodies, inhibition of binary fission and inability of the aberrant bodies to transform into infectious elementary bodies, characterize *in vitro* persistence. Transcriptomic and proteomic analyses have confirmed that there is continued genome replication and messenger RNA synthesis in the aberrant bodies, but altered cell division (Nicholson et al., 2003; Mäurer et al., 2007).

Although the persistence of *C. trachomatis* and *C. pneumoniae in vitro* is rather well studied, much less is known, if the features typical of *in vitro* persistent infection also characterize persistent experimental or human chlamydial infection. Recently, aberrant bodies were shown to develop in uterine horns of mice infected with *C. muridarum* and treated with amoxicillin (Phillips Campbell et al., 2012). Amoxicillin treatment also reduced vaginal shedding of infectious EB while accumulation of the chlamydial pre—16s rRNA was unaffected (Phillips Campbell et al., 2012). This indicates that chlamydial persistent infection can occur *in vivo*. As the bacteria become dormant and persistent, they are suggested to evade host protective immune responses, although the immune system can be still be stimulated at low-level which may contribute to the development of immune-mediated pathology (Beatty et al., 1994; Phillips Campbell et al., 2012). Follow-up studies on human ocular and recurrent genital *C. trachomatis* infections have shown features common to persistent state, such as the frequent detection of chlamydial nucleic acid while the organisms can infrequently be cultured (Hudson et al., 1992; Dean et al., 2000). Clinical data also suggests that *C. pneumoniae* may persist for months after the initial infection (Grayston, 1992), and persistent lung infections despite of antimicrobial therapy have been reported (Falck et al., 1996; Miyashita et al., 2002). *C. trachomatis* DNA has been demonstrated by *in situ* hybridization in the fallopian tube tissue from infertile women (Campbell et al., 1993; Barlow et al., 2001). Significantly elevated IgA antibody levels considered suggestive of persistent infection were found among the currently or formerly smoking men compared to their non-smoking co-twins (von Hertzen et al., 1998). Moreover, electron micrographs have demonstrated different stages of *C. pneumoniae* in atherosclerotic plaques (Spagnoli et al., 2007; Bobryshev et al., 2008), and antigens whose expression is up regulated during the persistent state of *C. pneumoniae* have been detected in human atheromas (Borel et al., 2012).

*In vitro*, chlamydial persistence has also been characterized at molecular level. Although the phenotype of persistent infections is rather similar irrespective of the inducer, the transcriptional studies on persistent chlamydial infection have failed to reveal a profile common for different models (Hogan et al., 2003; Goellner et al., 2006; Klos et al., 2009). This might reflect variation in the experimental systems but it could also indicate variability of the mechanisms acting in the different models of persistent infection. Based on the relative chlamydial rRNA transcript levels in persistently infected monocytes and actively growing epithelial cells, the metabolic rate of *C. trachomatis* in monocytes is lower than in the cells where *C. trachomatis* grows productively (Gérard et al., 2002). When chlamydial transcription and translation were analyzed upon IFN-γ exposure in the epithelial cells, the global chlamydial transcription was shown to be up regulated but the protein synthesis was reduced suggesting uncoupling of transcription and translation (Ouellette et al., 2006). Although this phenomenon can represent a successful survival strategy for *Chlamydiae*, it might also mean less potential chlamydia-derived biomarkers available for detection of the persistent stage. Nevertheless, quantitative proteomic profiling of the persistent and the acute chlamydial infection to identify differentially expressed proteins represents a possibility for providing diagnostic targets for the persistent infection. The proteins associated with persistent infection could be useful as biomarkers for the diagnosis, as therapeutic targets, and ultimately as response-to-therapy markers. However, the initial studies in this area also failed to reveal a common profile between different models and different chlamydial species (Mukhopadhyay et al., 2006). Upon exposure to IFN-γ, the presence of aberrant *C. trachomatis* forms was associated with down-regulation of the outer membrane proteins (OmpA, OmpB) in cell culture, while the expression of chlamydial GroEL was unaltered (Beatty et al., 1993). Attempts to characterize the protein composition of *C. pneumoniae* during persistent stage have shown a marked up-regulation of OmpA and GroEL upon IFN-γ exposure, whereas no significant decrease in bacterial protein expression was observed (Molestina et al., 2002). On the other hand, upon iron limitation unchanged levels of *C. pneumoniae* OmpA and GroEL were observed, while altogether twenty differentially regulated chlamydial proteins were observed (Wehrl et al., 2004). Many of these proteins remained unidentified.

A serum-based assay would be most desirable for the diagnosis of persistent infection. Serum samples are relatively easily obtained whereas tissue samples from the site of persistent infection often require invasive sampling and are thus not readily available. The recent studies on serology during persistent infection suggest that the antibody response could indeed reflect altered protein of chlamydial expression *in vivo* but this needs further studies (see below). Despite of all the recent discoveries, the development of diagnostic tests for persistent infections remains challenging.

In this minireview, diagnostic options of human persistent chlamydial infection will be discussed (see also Table 1).

**Table 1:** **Potential biomarkers of persistent human chlamydial infection**.

**Marker**	**Chlamydial species**	**Remarks**	**Condition**	**References**
Chlamydial LPS-containing immune complexes in serum	*C. pneumoniae*	Technically demanding assay	Acute myocardial infarction	Leinonen et al., 1990
			Chronic coronary heart disease	Linnanmäki et al., 1993
			COPD	Von Hertzen et al., 1997
			Stroke	Tarnacka et al., 2002
Chlamydial LPS in tissues/serum	*C. pneumoniae*	Technically demanding assay	Atherosclerosis	Kuo et al., 1993; Vikatmaa et al., 2010
			Acute coronary event	Tiirola et al., 2007
			Novel cardiovascular event	Pesonen et al., 2009a,b
Elevated IgG and IgA antibody	*C. pneumonia*		Asthma	Von Hertzen et al., 1997; Hahn et al., 2000
	*C. trachomatis*		Infertility	Sarov et al., 1986
IgA antibody response to whole bacteria	*C. pneumoniae*	Interlaboratory variation in MIF	Acute coronary syndrome	Huittinen et al., 2003; Miya et al., 2004
			Asthma	Sävykoski et al., 2004; Von Hertzen et al., 1997; Hahn and Saikku, 1995
			Predictor of treatment response	Hahn et al., 2006
			Increased intima-media thickness in children	Volanen et al., 2006
IgE antibody response	*C. pneumoniae*	Immunoblot format	Asthma, severity of asthma	Emre et al., 1995; Hahn et al., 2000, 2012
Chlamydial DNA in mucosal swabs or tissues	*C. pneumoniae*	Not detected in veins or in the vessel wall in the absence of pathology	Atherosclerosis	Kuo et al., 1993; Rosenfeld and Campbell, 2011
			COPD	Von Hertzen et al., 1997
	*C. trachomatis*		Trachoma	Mabey and Solomon, 2003
			Tubal factor infertility	Campbell et al., 1993; Barlow et al., 2001
Chlamydial rRNA in tissues	*C. trachomatis*	Denotes presence of metabolically active bacteria	Reactive arthritis	Gérard et al., 1998
			Trachoma	Burton et al., 2006
Antibody to GroEL (CPn0134 or CT110)	*C. pneumoniae*	Autoimmunity manifested as antibody to human Hsp60 contributes to pathogeneses	Asthma	Huittinen et al., 2001
			Coronary heart disease	Huittinen et al., 2002; Mahdi et al., 2002; Biasucci et al., 2003; Pesonen et al., 2009a
	*C. trachomatis*		Trachoma	Skwor et al., 2010
			Subfertility	Karinen et al., 2004
			Infertility	Linhares and Witkin, 2010
			Tubal factor infertility	Tiitinen et al., 2006; Bunk et al., 2008; Budrys et al., 2012
Antibody to CT858	*C. trachomatis*		Trachomatous trichiasis	Skwor et al., 2010
Seroresponse to a panel consisting of CT110, CT376, CT557, CT443 and absence of response to CT875, CT147	*C. trachomatis*	Some antigens also recognized by sera from acute infection Remains to be confirmed in larger settings	Tubal factor infertility	Budrys et al., 2012
Seroresponse to a panel consisting of antigens CPn0695, CPn0134, CPn0626CPn0702, CPn 449/450, CPn 0854,CPn 0963, CPn1016	*C. pneumoniae*	Remains to be confirmed in larger settings	Presence of *C. pneumoniae* DNA in the coronary artery or in the PBMC	Bunk et al., 2008

## Culture

By definition, the detection of persistent chlamydial infection (defined as the presence of viable but non-cultivable chlamydiae) by culture is not possible. Culture has, however, been successfully used in the early studies on persistency. During experimental mouse infection, *Chlamydiae* are able to remain in tissues after cessation of shedding and the infection can be reactivated by cortisone treatment (Yang et al., 1983; Malinverni et al., 1995; Laitinen et al., 1996; Cotter et al., 1997). In these studies, mice were infected and the infection was followed by culture. After the clearance of a culture-positive infection, cortisone was administered. This obviously activated persistent *Chlamydiae*, as infectious bacteria could again be recovered, at least in a proportion of mice.

Although the persistent state is still rather poorly defined *in vivo*, this observation is of potential significance in human persistent infection: Corticosteroids are included e.g., in the treatment of patients with exacerbations of asthma and chronic obstructive bronchitis, conditions associated with persistent *C. pneumoniae* infection (Hahn et al., 1991), and the steroid therapy could reactive infection. If this takes place *in vivo*, it might aggravate infection, and enhance transmission but on the other hand, it might make the bacteria more sensitive to action of antibiotics. There is, however, little, if any clinical data to support this. Partly this could be due to issues related to proper sample collection, as the persistent chlamydial forms can localize in tissues, and not necessarily in the mucosal surface available for swabbing (Cappuccio et al., 1994). Recently, biologic response modifiers have been introduced to the treatment of inflammatory diseases. Whether these new therapies could also reactivate persistent chlamydial infection, remains to be studied.

## Antigen detection

To circumvent limitations of the culture, immunological antigen detection methods, such as enzyme immunoassay and direct immunofluorescence staining were developed for diagnosis of chlamydial infections. Theoretically, detection of a chlamydial antigen in clinical specimens could serve as a diagnostic test for persistent infection. However, the general notion is that the detection of chlamydial EB in mucosal smears indicates presence of a current *C. trachomatis* (Havlichek et al., 1990) or *C. pneumoniae* (Grayston et al., 1986) infection. It would obviously require more invasive sampling than swabbing of the mucosa to demonstrate the hallmarks of persistent infection, the aberrant bodies, at the site of infection. Moreover, the amount of aberrant bodies in the specimen is likely to be very small and reagents to visualize them are not readily available.

For research purposes, the detection of chlamydial antigen(s) by immunocytochemistry (ICC) in tissues has proven useful and ICC has assisted studies on clinical features of persistent infections, including post infectious tubal infertility (Patton et al., 1994) and atherosclerosis (Kuo and Campbell, 2000). Instead, detection of a soluble circulating chlamydial antigen could represent a practical tool in diagnosis. The quantification of chlamydial lipopolysaccharide (cLPS) in human sera by enzyme immunoassay represents a potential marker for persistent infection (Tiirola et al., 2006). Chlamydial LPS was detected in serum during acute coronary events and its presence correlated with the C-reactive protein levels (Tiirola et al., 2007). Moreover, the circulating cLPS was associated with a new cardiovascular event during the follow-up period (Pesonen et al., 2009b). Also, circulating chlamydial LPS-containing immune complexes have been suggested to be important in the pathogenesis and serve as markers of persistent infection (Leinonen et al., 1990; Linnanmäki et al., 1993), but the assay is technically rather demanding and it has only been used in research.

## Nucleic acid amplification

Nucleic acid amplification test kits (NAATs) have revolutionized the diagnosis of acute *C. trachomatis* infections offering high sensitivity and specificity. The NAATs, including polymerase chain reaction (PCR), are also well suitable for the detection of persistent chlamydial infection, when lower quantities of *Chlamydiae* are produced challenging the sensitivity of the detection method. Indeed, the use of amplification technologies and culture has given us clues that persistent, non-cultivable chlamydial infections do occur *in vivo*. Trachoma in children can often be diagnosed by culture, whereas the agent can no longer been cultured in the blinding stage (Grayston and Wang, 1975). Similarly, *C. trachomatis* can be cultured in acute lower genital tract infections, while culture rarely succeeds in upper genital infections, including tubal factor infertility, although *C. trachomatis* DNA can be amplified by PCR (Brunham et al., 1985). The mere demonstration of bacterial DNA does not, however, indicate viability, but could be related to the higher sensitivity of the PCR method *per se*. Identification of chlamydial rRNA primary transcripts indicates that the bacteria are metabolically active (Gérard et al., 1997). Indeed, in the synovial samples from patients with reactive arthritis primary rRNA transcripts and mRNA from chlamydial genes were present (Gérard et al., 1998).

PCR with *C. pneumoniae*-specific primers has played an important role in confirming the presence of DNA from these bacteria in atheromas and circulating blood cells. In combination with serology and ICC, the PCR has been a valuable tool in the studies confirming the link between atherosclerosis and infections, especially with persistent *C. pneumoniae* infection (Campbell and Kuo, 2004; Rosenfeld and Campbell, 2011). Current data supports that this condition represents a persistent infection: *Ex vivo* culture of *C. pneumoniae* from atherosclerosis is rare, while PCR can frequently detect chlamydial DNA in diseased tissues (Campbell and Kuo, 2004). Studies in mice show that the presence of *C. pneumoniae* DNA in tissues is an indicator of prior infection, and when the bacteria are not cultivable it is suggestive of persistence, as DNA in the non-viable bacteria degrades fast *in vivo* (Moazed et al., 1998).

## Serology

Serology has been a valuable tool in epidemiological studies, in studies describing clinical spectrum of chlamydial infections, including persistent infection, and in research. Serology is also commonly used to diagnose acute *C. pneumoniae* infections in clinical practice while its value in the laboratory diagnosis of acute *C. trachomatis* infections is slim. It may, however, prove helpful when studying complications of the acute phase (e.g., reactive arthritis) and manifestations of persistent infections (e.g., tubal factor infertility). Microimmunofluorescence (MIF) test using fixed purified whole bacteria as antigen has been considered a golden standard of chlamydial serology, but automatable enzyme immunoassays (EIA) have taken root in clinical laboratories, partly because the MIF can suffer from subjectivity in interpretation and interlaboratory variation (Peeling et al., 2000). The MIF and most EIAs detect antibody response against proteins on the surface of *Chlamydiae*, and established guidelines on the diagnostic criteria for acute *C. pneumoniae* infections are available: IgM appears in the first infection in 3 weeks, IgG in 6–8 weeks, and if adequately timed paired sera are available, a ≥4-fold IgG titer rise can be seen upon infection and reinfection (Wang and Grayston, 1986).

Despite of considerable efforts, persistent infections are difficult to diagnose, and no widely accepted serological criteria for persistent infection exist at present. IgG antibody can persist long after the acute phase (Puolakkainen et al., 1986; Paldanius et al., 2005), and only reflects (previous) exposure to the organisms. Some markers have yet been brought forward. Elevated IgE levels are almost always associated with asthma, a proposed manifestation of persistent *C. pneumoniae* infection, but the inducing factor for the IgE response has largely remained unknown. In a recent case-control study, *C. pneumoniae*-specific IgE antibody response was strongly associated with asthma and with severity of asthma in adults (Hahn et al., 2012). IgA antibodies are naturally short-lived with a half-life of 5–6 days. Consistently present/elevated *C. pneumoniae* IgA antibody titers (Saikku et al., 1992), and elevated serum *C. pneumoniae* IgA and IgG antibodies together with elevated C-reactive protein (Huittinen et al., 2003; Miya et al., 2004; Sävykoski et al., 2004) have been proposed as serological markers of persistent *C. pneumoniae* infection. IgA response could also be a predictor of treatment response (Hahn et al., 2006). Additionally, an association between *C. pneumoniae* IgA and human Hsp60 antibodies noted in patients with risk of coronary events (Huittinen et al., 2002) suggests that antibodies to human Hsp60 could be induced by bacterial GroEL (*C. pneumoniae* Hsp60 homolog, CPn0134) during persistent infection. Likewise, enhanced antibody response to *C. trachomatis* EB and chlamydial GroEL (*C. trachomatis* Hsp60 homolog, CT110) protein is associated with trachoma (Skwor et al., 2010) subfertility (Karinen et al., 2004), infertility, especially tubal factor infertility, a manifestation of persistent *C. trachomatis* infection (Toye et al., 1993; Witkin et al., 1998; Tiitinen et al., 2006).

Although the performance of the serological assays using whole bacteria as antigen and developed for the detection of acute infection might not be perfect, they have been valuable in epidemiological studies and when patient populations with a given condition have been studied. Moreover, the expanding clinical spectrum of chlamydial diseases, including those associated with persistent infection, was initially based on the findings obtained with the traditional but specific chlamydial serology. The antibodies recognized by the MIF are directed against the surface proteins of the chlamydial EB. OmpA (major outer membrane protein) is a dominant immunogen in *C. trachomatis*, but the (*C. pneumoniae*) proteins contributing to the MIF reactivity have largely remained unknown (Bunk et al., 2008). Proteomic analyses of chlamydial infection have revealed that a part of the surface proteins are more frequently and a part of them less frequently expressed during persistent than acute infection. Consequently, the MIF test as well as the EIAs using whole bacterial antigen might not be able to adequately discriminate between acute and persistent infections (Bunk et al., 2008). In support of this observation, reactivity of the sera from individuals with persistent infection in the MIF tests is necessarily not reflected by the reactivity of these sera with differentially expressed antigens, including chlamydial GroEL (Huhtinen et al., 2001; Bunk et al., 2008).

Proteomic profiling of chlamydial infection may enable the development of more precise serological assays that could better differentiate responses related to acute infection, cured past infection (serological scar) and persistent infection. The history and clinical status of the patients donating the serum specimens must be characterized appropriately. This is not always possible and straightforward, and may need invasive and extensive examinations. To identify antibody response patterns associated with persistent *C. pneumoniae* infection, Bunk et al. (2008) applied a proteomic approach with 2D-gel electrophoresis combined with immunoblotting. Using serum specimens from patients with probable persistent infection, based on the presence of *C. pneumoniae* DNA in their coronary arteries or in their PBMC, and blood donors without such markers of persistent infection, the researchers could identify a differential response against 12 *C. pneumoniae* antigens, including both increased and decreased reactivity (Bunk et al., 2008). Sera from the PCR-positive individuals reacted more intensely with *C. pneumoniae* CPn0695 (OmpA) and CPn0134 (GroEL) [and also with 6 other antigens, CPn0626 (RpoA), CPn0702 (YscC), CPn449/450 (Pmp10), CPn0854 (PorB), CPn0963 (Pmp21), and CPn1016 (Cpaf)] than sera from controls. The observed antibody response pattern was in accordance with the earlier mentioned *in vitro* proteomic studies showing higher and lower seroreactivity toward the proteins that were shown to be up-regulated and down-regulated, respectively, during IFN-γ-mediated persistence (Molestina et al., 2002; Mukhopadhyay et al., 2006). A recent description of a *C. pneumoniae* ORFeome library covering 99% of the 1052 the ORFs of *C. pneumoniae* provides an additional novel tool for studying the seroresponses during persistent phase in-depth (Maier et al., 2012).

To identify novel *C. trachomatis* proteins or protein combinations associated with different stages of infection, a high-resolution whole genome scale protein array (Wang et al., 2010) has been used to profile serological responses. Sera from patients with tubal factor infertility (TFI) and their controls recognized many *C. trachomatis* proteins (Wang et al., 2010). Comparison of the antibody profiles revealed that the sera from TFI patients preferentially recognized 30 *C. trachomatis* proteins. Those included CT110 (GroEL; 71% of sera reacted with this protein) confirming the earlier findings that response to the Hsp60 homolog is associated with TFI. In this study, no single antigen yielded 100% specificity and >50% sensitivity for detection of TFI, whereas a combination of CT443 (omcB) and CT381 (arginine binding protein) antigens yielded the highest detection sensitivity (68%) for chlamydial TFI while maintaining 100% specificity (Rodgers et al., 2011). However, also *C. trachomatis* antibody-positive individuals with acute *C. trachomatis* infection frequently reacted with GroEL, and diminished the value of this antigen as a marker of persistent infection. Subsequently, panels of *C. trachomatis* antigens that could predict tubal pathology and acute infection have been suggested. Using the same high-resolution approach, reactivity with four proteins [CT110 (GroEL), CT376 (malate dehydrogenase), CT557 (dihydrolipoamide hydrolase), and CT443 (OmcB)] could distinguish women with TFI from fertile women with a sensitivity of 63% and specificity of 100%. Reactivity with a combination of two *C. trachomatis* antigens (CT875 and CT147, both coding for hypothetical proteins) could discriminate between women with acute infection and those with TFI (Budrys et al., 2012) enabling sequential screening to identify patients with these conditions.

## Conclusion

A serum-based assay (either for antibody detection, antigen detection or nucleic acid amplification) would be desirable for the detection of persistent infection. Serum samples are relatively easily obtained whereas tissue samples from the site of persistent infection are not readily available. The above-mentioned recent studies utilizing proteomics and bacterial genome wide approaches by Bunk et al. (2008), Rodgers et al. (2011) and Budrys et al. (2012) indicate that chlamydial serology can be made more precise and refined. These studies also demonstrate the first evidence of differential serological response as a function of the infection status. Discrimination of antibody response related to persistent infection from that related to acute infection and from a serological scar is important and clinically relevant, and these promising approaches wait to be confirmed in studies with larger populations.

### Conflict of interest statement

The author declares that the research was conducted in the absence of any commercial or financial relationships that could be construed as a potential conflict of interest.
